# Estimation of the Radiographic Parameters for Hallux Valgus From Photography of the Feet Using a Deep Convolutional Neural Network

**DOI:** 10.7759/cureus.65557

**Published:** 2024-07-28

**Authors:** Kana Inoue, Satoshi Maki, Satoshi Yamaguchi, Seiji Kimura, Ryuichiro Akagi, Takahisa Sasho, Seiji Ohtori, Sumihisa Orita

**Affiliations:** 1 Department of Medical Engineering, Graduate School of Science and Engineering, Chiba University, Chiba, JPN; 2 Department of Orthopedic Surgery, Graduate School of Medicine, Chiba University, Chiba, JPN; 3 Center for Frontier Medical Engineering, Chiba University, Chiba, JPN; 4 Graduate School of Global and Transdisciplinary Studies, College of Liberal Arts and Sciences, Chiba University, Chiba, JPN; 5 Center for Preventive Medical Sciences, Chiba University, Chiba, JPN; 6 Department of Orthopedic Surgery, Chiba University, Chiba, JPN

**Keywords:** photograph, artificial intelligence, deep learning, hallux valgus, foot

## Abstract

Background: Hallux valgus (HV), also known as bunion deformity, is one of the most common forefoot deformities. Early diagnosis and proper evaluation of HV are important because timely management can improve symptoms and quality of life. Here, we propose a deep learning estimation for the radiographic measurement of HV based on a regression network where the input to the algorithm is digital photographs of the forefoot, and the radiographic measurement of HV is computed as output directly. The purpose of our study was to estimate the radiographic parameters of HV using deep learning, to classify the severity by grade, and to assess the agreement of the predicted measurement with the actual radiographic measurement.

Methods:There were 131 patients enrolled in this study. A total of 248 radiographs and 337 photographs of the feet were acquired. Radiographic parameters, including the HV angle (HVA), M1-M2 angle, and M1-M5 angle, were measured. We constructed a convolutional neural network using Xception and made the classification model into the regression model. Then, we fine-tuned the model using images of the feet and the radiographic parameters. The coefficient of determination (R^2^) and root mean squared error (RMSE), as well as Cohen's kappa coefficient, were calculated to evaluate the performance of the model.

Results: The radiographic parameters, including the HVA, M1-M2 angle, and M1-M5 angle, were predicted with a coefficient of determination (R^2^)=0.684, root mean squared error (RMSE)=7.91; R^2^=0.573, RMSE=3.29; R^2^=0.381, RMSE=5.80, respectively.

Conclusion: The present study demonstrated that our model could predict the radiographic parameters of HV from photography. Moreover, the agreement between the expected and actual grade of HV was substantial. This study shows a potential application of a convolutional neural network for the screening of HV.

## Introduction

Hallux valgus (HV), also known as bunion deformity, is one of the most common forefoot deformities and is characterized by a medial deviation of the first metatarsal bone, a lateral deviation of the hallux, and a prominent metatarsal head [1]. The deformity is associated with disabling pain leading to significant morbidity and quality of life issues. The prevalence of HV increases with age, affecting 23% of adults aged 18-65 and 36% of adults over 65. HV is more prevalent in women, and women are diagnosed two to 15 times more often than men [2,3].

﻿Early diagnosis and proper evaluation of HV are important because timely management can improve symptoms and quality of life. The most commonly used reference standard measurements for assessing HV are the HV angle (HVA) and the inter-metatarsal angle on foot radiographs. However, radiographs are not the best screening tool due to their availability, cost, and radiation exposure. Instead of radiographs, more accessible and less invasive assessments have been reported as alternatives, including visual categorical grading scales to assess the extent of deformity in HV [4-6] and quantitative measurement of the hallux valgus angle using digital photographs [7]. Visualization of cosmetic appearance improvements has been reported to be associated with patient perception of subjective pain and improved function, highlighting the importance of digital photography [8,9]. Although it is feasible to determine the severity of HV to some extent by the appearance of the foot, the limitation is that this requires training and experience for the examiner, and is not automated.

Deep learning is a type of artificial intelligence that has been extensively applied in the medical imaging field in recent years. The applications of convolutional neural networks (CNNs) to medical imaging include classification, object detection, and semantic segmentation [10-12]. Here, we propose a deep learning estimation for the radiographic measurement of HV based on a regression network where the input to the algorithm is digital photographs of the forefoot and the radiographic measurement of HV is computed as output directly. The purpose of our study was to estimate the radiographic parameters of HV from photographs of the foot using deep learning, to classify the severity by grade, and to assess the agreement of the predicted measurement with the actual radiographic measurement.

## Materials and methods

Patients

The present study was conducted using secondary data from the previous prospective study. The study was approved by the local institutional review board of the Graduate School of Medicine, Chiba University. The additional requirement for informed consent was waived by the local institutional review board of the Graduate School of Medicine, Chiba University, because of the retrospective analysis. All procedures involving human participants were in accordance with the 1964 Declaration of Helsinki and its later amendments. Patients who visited our foot and ankle clinic from February to June 2016 and underwent weight-bearing dorsal X-rays of the foot were recruited. Patients with acute inflammatory diseases such as cellulitis and gout, or with a history of ankle surgery, fractures, or dislocation within the past year, were excluded. There was a total of 131 patients enrolled in this study.

Photography of the feet

**Figure 1:**
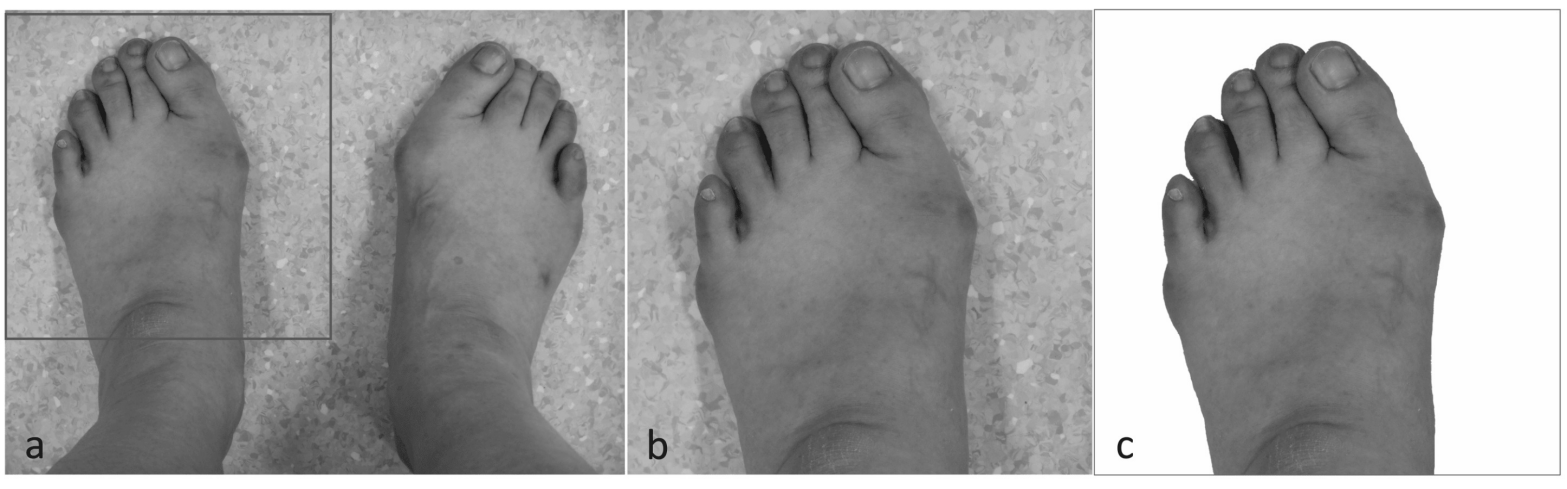
Image preprocessing for the convolutional neural network model training and validation. An original photograph of the feet (a). The images of the feet were divided into right and left and cropped to a minimum region, which included the ankle to the toes (b). The background was removed semi-automatically using PowerPoint (c).

Patients took photographs and also received radiographs during their outpatient visits. Patients photographed their feet using a digital camera or smartphone according to an instruction sheet, which was given to each subject to standardize the foot position for the photograph. The camera or smartphone type was not specified. Participants who did not have a camera or smartphone were provided with a digital camera (IXY 150, Canon, Ota Ward, Tokyo). Participants did not receive additional instructions or assistance from the research staff. This was to simulate a situation where a patient uses a smartphone app to take a picture of his or her foot to be diagnosed with HV. The images of the feet were divided into right and left and cropped to a minimum region, which included the ankle to the toes (Figures 1a, 1b). The background was removed semi-automatically using PowerPoint (Microsoft Corporation, Redmond, WA) (Figure 1c). In order to correctly identify the big toe and little toe, the photo of the left foot was flipped horizontally so that all foot orientations were recognized as the right foot. A total of 346 images of feet were acquired. One hundred and seventeen patients took images of both feet, and 43 of the 117 were taken twice. In addition, 14 patients took pictures of one foot, and three out of 14 took images twice.

Radiographic dataset

**Figure 2:**
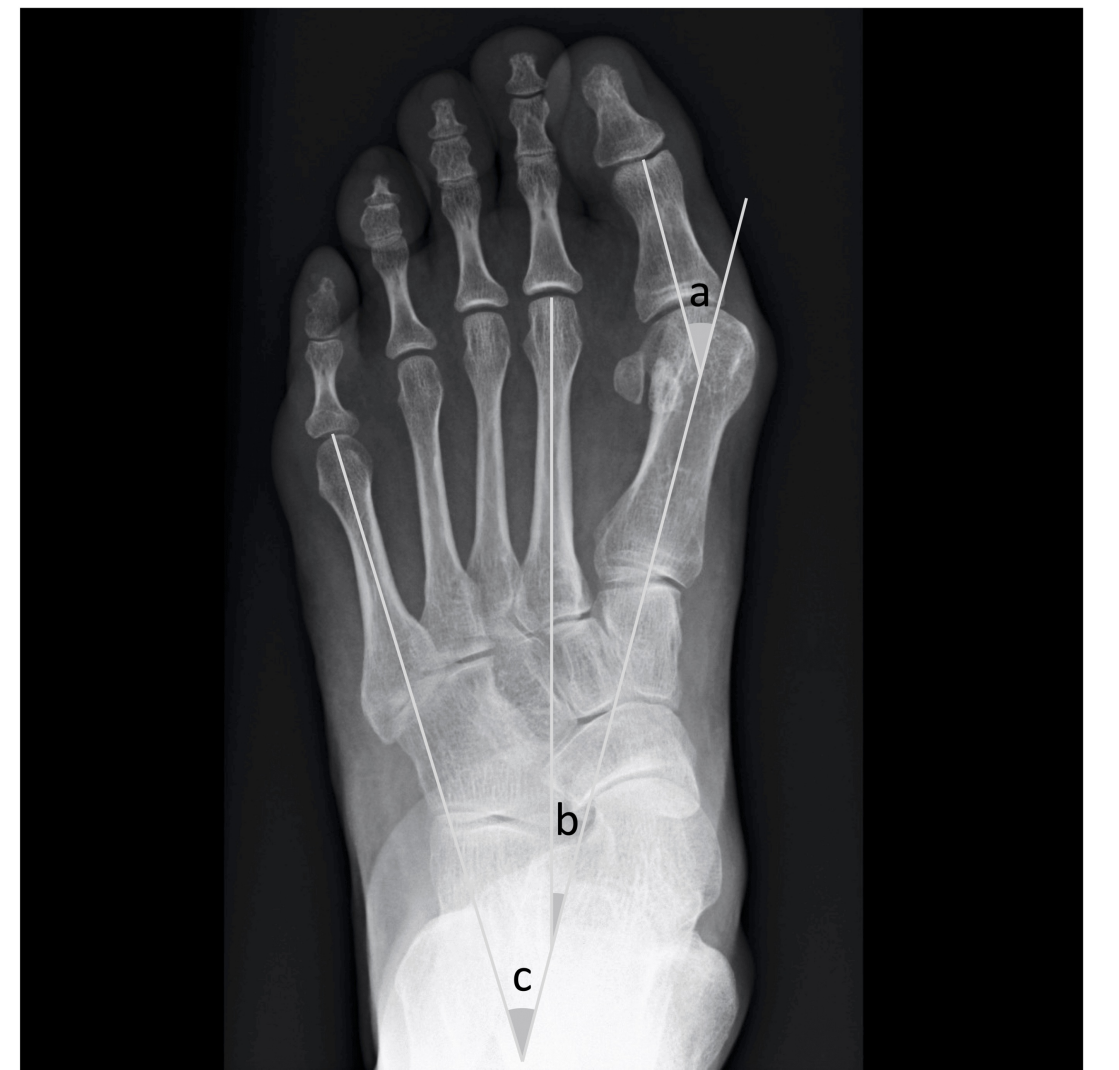
The radiographic parameters of HV, including the HVA, the M1-M2 angle, and the M1-M5 angle. The HVA refers to the angle, which is formed by the axis of the proximal phalanx of the hallux and the axis of the first metatarsal (a). ﻿The M1-M2 angle is the angle, which is formed by the longitudinal axis of the first and second metatarsals (b). The M1-M5 angle is the angle, which is formed by the longitudinal axis of the first and fifth metatarsals (c). HV: hallux valgus, HVA: HV angle.

For the weight-bearing, dorsoplantar-view radiographs of the feet, patients were instructed to stand in a relaxed position, distribute the weight evenly on both feet and keep the feet parallel. The central beam is angled to approximately 15-20 degrees towards the heel at a distance of 100 cm, directly parallel to the long axis of the foot, and centered on the second tarsometatarsal joint [13]. The radiographic parameters of HV, including the HVA, M1-M2 angle, and M1-M5 angle, were measured. The HVA refers to the angle formed by the axis of the proximal phalanx of the hallux and the axis of the first metatarsal (Figure 2a). ﻿The M1-M2 angle is the angle formed by the longitudinal axis of the first and second metatarsals (Figure 2b). The M1-M5 angle is the angle formed by the longitudinal axis of the first and fifth metatarsals (Figure 2c). Hallux valgus, defined as HVA of ≥20°, was classified as mild (20°, 30°), moderate (30°, 40°), or severe (>40°) [14].

HV was measured using the angular measurement function of the picture archiving and communication system and rounded to the nearest whole number for analysis. All images were measured by a board-certified orthopedic surgeon (SM 14 years of experience). A total of 248 radiographs were taken, with 117 patients taking radiographs of both feet and 14 people taking radiographs of one foot.

CNN model construction

The CNN architecture was constructed using Python 3.6.7 (Python Software Foundation, Wilmington, USA) and Keras 2.2.4 with Tensorflow 2.0.0 at the backend. The models were separately constructed for the HVA, M1-M2 angle, and M1-M5 angle. In this study, we adapted the Xception architectural model, which had been previously trained using images with ImageNet [15,16]. ﻿The input images were resized to 299 × 299 pixels. We replaced the final layer of the model with a global average pooling layer and a fully connected layer to make the classification model into a regression model [17]. Then, we fine-tuned the pre-trained model using photographs of the feet and the measured radiographic parameters. The first 26 layers were frozen, and the weights were not modified during the training process. The rest of the layers were retrained with our data. The network was trained over 1000 epochs with a learning rate of 0.1, which was reduced if no improvement was seen. Adam was used for the optimizer, and the root mean squared error (RMSE) was used for the loss function. The data augmentation was done by ImageDataGenerator including a rotation angle range of 90°, a width shift range of 0.1, a height shift range of 0.1, a shear range of 0.1, and a horizontal flip of 50%. The CNN was trained and validated using a computer with a GeForce RTX 2060 graphics processing unit (NVIDIA, Santa Clara, CA).

Performance evaluation of the model

To train and evaluate the CNN model, we performed a five-fold cross-validation. Photographs of the foot were randomly divided into five equal-sized independent subgroups. Images taken from the same patients were assigned to the same subgroup. In each iteration, data from the four subgroups were selected as a training set, and the remaining independent subgroups served as validation data. In the validation phase, the performance to predict the radiographic parameters was assessed using the remaining independent subgroup. This cross-validation process was repeated five times. The coefficient of determination (R^2^) and the RMSE were calculated to evaluate the performance of the CNN using the sklearn.metrics.r2_score and the square root of sklearn. metrics.mean_squared_error function from the Scikit-learn library (version 0.23.2), respectively. The severity of hallux valgus was graded as normal, mild, moderate, and severe [14] from the degree of the predicted HVA, and the agreement with the severity grading based on the radiographic HVA measurement was evaluated using Cohen's kappa coefficient. The Cohen's kappa coefficient was calculated using sklearn. metrics.cohen_kappa_score function from the Scikit-learn library.

## Results

Baseline patient characteristics

Table 1 shows the baseline characteristics and radiographic data of the participants.

**Table 1:** Baseline characteristics of the patients.

Variable	Patients (n=131)
Age, y	61.2 ± 13.3
Sex, n	
Male	34
Female	97
Laterality, n	
Right	123
Left	125
Radiographic parameters, °	
Hallux valgus angle	26.2 ± 16.2
M1-M2 angle	13.9 ± 5.1
M1-M5 angle	32.8 ± 7.0

Model performance

**Figure 3:**
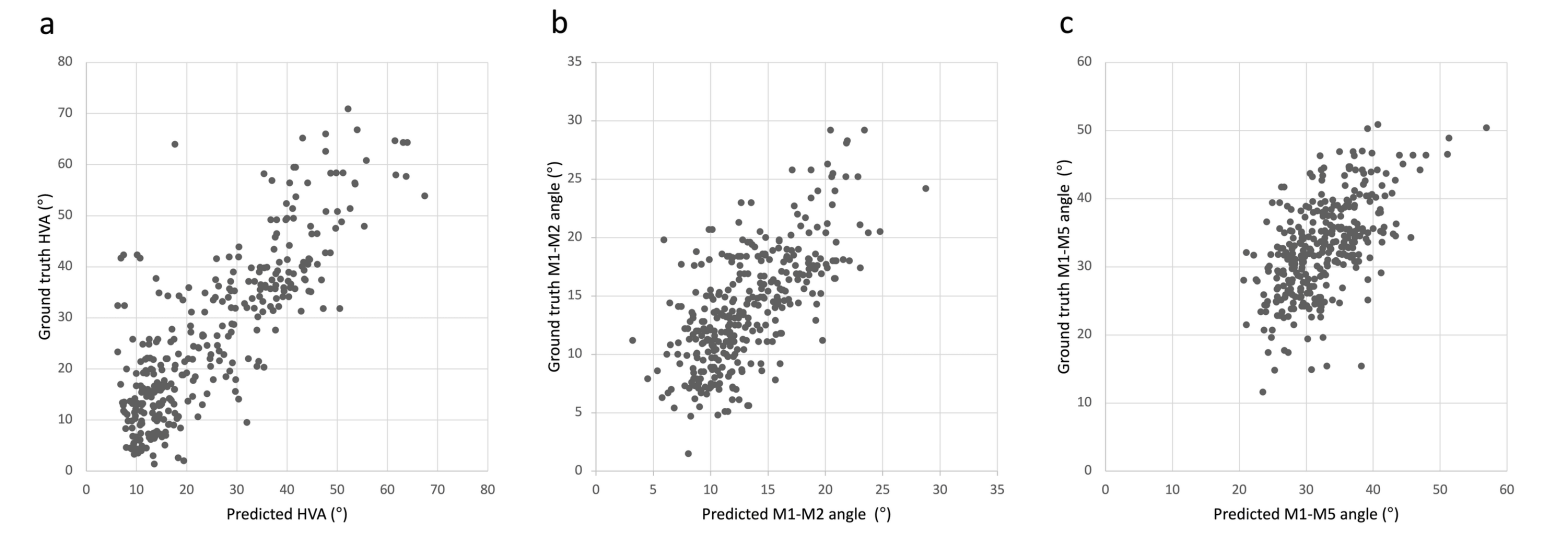
A scatter plot of the ground truth vs. predicted HVA (a), M1-M2 angle (b), and M1-M5 angle (c). HVA: hallux valgus angle.

The radiographic parameters of hallux valgus, which included the HVA, M1-M2 angle, and M1-M5 angle, were predicted with R^2^=0.684, RMSE=7.91; R^2^=0.573, RMSE=3.29; R^2^=0.381, RMSE=5.80, respectively. Agreement between the predicted and ground truth radiographic parameters was substantial for the HVA but was only fair for the M1-M2 and M1-M5 angles. Scatter plots of the predicted radiographic parameters versus the ground truth are shown in Figures 3a-3c. The confusion matrices of the CNN model to predict the severity grade of HV are indicated in Table 2. The Cohen’s weighted κ coefficient for agreement between the predicted and actual severity grade of HV was 0.809, indicating substantial agreement.

**Table 2:** The relationship between the ground truth and the predicted severity grade of HVA. HVA: hallux valgus angle.

	Predicted severity grade of HVA				
Ground truth severity grade of HVA		Normal	Mild	Moderate	Severe
	Normal	132	12	2	0
	Mild	24	25	6	0
	Moderate	7	17	33	16
	Severe	5	2	13	43

## Discussion

The present study demonstrates that a CNN can predict the radiographic parameters of HV from photographs. Moreover, the agreement between the predicted HV grade by the CNN and the actual grade was substantial. This study indicates a potential application of a CNN for the screening of HV.

The three radiographic parameters of HV, namely, the HVA, M1-M2 angle, and M1-M5 angle, were estimated from photography using CNN. The predicted HVA showed substantial agreement with the ground truth, and the predicted M1-M2 and M1-M5 angles showed fair agreement with the ground truth. Several studies have attempted to predict the HV angle from a photograph of the foot. Nix et al. measured the hallux valgus angle quantitatively using digital photographs and showed that the measurement was reliable and highly correlated with the radiographic measurement (Pearson’s r=0.96) [18]. However, in that study, the photographs were taken by an experienced rater under strict parameters to ensure that the position and angle of the camera matched perfectly with those from the radiography. Yamaguchi et al. demonstrated that the photographic HVA, which is the angle between the two lines drawn on the medial side of the hallux and the medial side of the foot on a photograph, was reproducible and useful as a non-radiographic measurement to quantify HV deformity [7]. ﻿Moreover, the photographic HVA showed a strong correlation with the HVA measured on the radiograph (R^2^=0.891, P<0.001) [7]. Although the agreement between the HVA predicted by the CNN and the HVA measured on the radiograph is not as good as the agreement found in the previous two studies, the strength of the present study is that the prediction is automatic and does not require expertise or experience for either the acquisition of the photograph or the rater. Theoretically, this could enable remote screening for HV, without necessitating an initial office visit.

The HV grade based on the HVA predicted by the CNN was substantially associated with the HV grade determined by radiographic measurement (Cohen’s weighted κ coefficient=0.809). There have been a few reports of grading hallux valgus based on the appearance of the foot using photographs [4-6]. Menz et al. reported that the Manchester scale, consisting of four standardized photographs of the feet, was ﻿highly correlated with the HVA (Spearman’s ρ=0.73, P<0.01), and moderately associated with the M1-M2 angle (Spearman's ρ=0.49, P<0.01) obtained from radiographs [5]. The Manchester scale showed ﻿high test-retest reliability, and the participants' self-rated Manchester scale was correlated strongly with scores obtained by expert raters [6]. ﻿The agreement between the observations of the participants and the expert raters was substantial (weighted kappa=0.71 to 0.80) [6]. Another self-report instrument for HV with a five-grade foot appearance, which weighted kappa scores (left and right feet combined), indicated 0.45 for the agreement between the participants and expert raters, 0.53 at one to two months and 0.51 at three to six months, for participant repeatability, and 0.82 for expert rater repeatability [4]. The agreement between the predicted HVA and the ground truth HVA in the present study was comparable to that of this previous study. Since the severity of the HV grade is generally evaluated in 10-15 degree increments, the HV grade predicted by the CNN is reliable enough to detect the difference in the HV grade given that the RMSE of the angle was 7.91. This study indicates the potential application of a CNN for the screening of hallux valgus. Using the CNN, the HV angles and HV grades can be estimated with substantial accuracy without requiring the experience of the examiner. Thus, this study could be the first step towards the application of a CNN using non-radiographic images for the screening of hallux valgus in the near future.

Several limitations should be noted in the present study. First, the dataset used in the study included 337 images of the foot, which is relatively small for a deep-learning study. A larger number of images for training could contribute to better agreement between the predicted and actual radiographic parameters. Second, we did not strictly match the position of the feet when taking radiographs and photographs. Third, the self-photography may vary across the participants because they just followed the instruction sheet and had no further assistance. However, this inconsistency in foot position or self-photography represents the clinical setting in which the CNN would be used.

## Conclusions

The CNN model was able to predict the radiographic parameters of HV from photography with fair to substantial agreement. Also, the agreement between the actual HV grades and the grades predicted by CNN was substantial. Our study demonstrates the potential application of a CNN for the screening of HV.

## References

[1] Wülker N, Mittag F (2012). The treatment of hallux valgus. Dtsch Arztebl Int.

[2] Nix S, Smith M, Vicenzino B (2010). Prevalence of hallux valgus in the general population: a systematic review and meta-analysis. J Foot Ankle Res.

[3] Piqué-Vidal C, Solé MT, Antich J (2007). Hallux valgus inheritance: pedigree research in 350 patients with bunion deformity. J Foot Ankle Surg.

[4] Roddy E, Zhang W, Doherty M (2007). Validation of a self-report instrument for assessment of hallux valgus. Osteoarthritis Cartilage.

[5] Menz HB, Munteanu SE (2005). Radiographic validation of the Manchester scale for the classification of hallux valgus deformity. Rheumatology (Oxford).

[6] Menz HB, Fotoohabadi MR, Wee E, Spink MJ (2010). Validity of self-assessment of hallux valgus using the Manchester scale. BMC Musculoskelet Disord.

[7] Yamaguchi S, Sadamasu A, Kimura S (2019). Nonradiographic measurement of hallux valgus angle using self-photography. J Orthop Sports Phys Ther.

[8] Bahar H, Yildiz KI (2021). Association of visual appearance on outcomes after hallux valgus surgery. Foot Ankle Int.

[9] Del Balso C, Taylor MA, Ching M, Lawendy AR, Sanders DW (2022). Preoperative photography improves patient satisfaction following hallux valgus surgery. Foot Ankle Surg.

[10] Galbusera F, Casaroli G, Bassani T (2019). Artificial intelligence and machine learning in spine research. JOR Spine.

[11] Yang S, Yin B, Cao W, Feng C, Fan G, He S (2020). Diagnostic accuracy of deep learning in orthopaedic fractures: a systematic review and meta-analysis. Clin Radiol.

[12] Kalmet PH, Sanduleanu S, Primakov S (2020). Deep learning in fracture detection: a narrative review. Acta Orthop.

[13] Tanaka Y, Takakura Y, Takaoka T, Akiyama K, Fujii T, Tamai S (1997). Radiographic analysis of hallux valgus in women on weightbearing and nonweightbearing. Clin Orthop Relat Res.

[14] Tachibana S (2014). Etiology, clinical condition, diagnosis (in Japanese). Hallux Valgus Practice Guide l.

[15] Chollet F (2017). Xception: Deep learning with depthwise separable convolutions. Comput Vis Pattern Recognit.

[16] Russakovsky O, Deng J, Su H (2014). ImageNet large scale visual recognition challenge. Int J Comput Vis.

[17] Toba S, Mitani Y, Yodoya N (2020). Prediction of pulmonary to systemic flow ratio in patients with congenital heart disease using deep learning-based analysis of chest radiographs. JAMA Cardiol.

[18] Nix S, Russell T, Vicenzino B, Smith M (2012). Validity and reliability of hallux valgus angle measured on digital photographs. J Orthop Sports Phys Ther.

